# Occurrence and genotypes of *Cryptosporidium* spp., *Giardia duodenalis*, and *Blastocystis* sp. in household, shelter, breeding, and pet market dogs in Guangzhou, southern China

**DOI:** 10.1038/s41598-020-74299-z

**Published:** 2020-10-20

**Authors:** Shenquan Liao, Xuhui Lin, Yongxiang Sun, Nanshan Qi, Minna Lv, Caiyan Wu, Juan Li, Junjing Hu, Linzeng Yu, Haiming Cai, Wenwan Xiao, Mingfei Sun, Guoqing Li

**Affiliations:** 1 Key Laboratory of Livestock Disease Prevention of Guangdong Province; Maoming Branch, Guangdong Laboratory for Lingnan Modern Agriculture; Scientific Observation and Experiment Station of Veterinary Drugs and Diagnostic Techniques of Guangdong Province, Ministry of Agriculture; Institute of Animal Health, Guangdong Academy of Agricultural Sciences, Guangzhou, 510640 Guangdong Province People’s Republic of China; 2 College of Veterinary Medicine, South China Agricultural University, Guangzhou, 510640 Guangdong Province People’s Republic of China

**Keywords:** Genotype, Sequencing, Evolution, Genetics, Health care, Risk factors

## Abstract

*Cryptosporidium* spp.*, Giardia duodenalis*, and *Blastocystis* sp. are common intestinal protozoans that infect humans and animals worldwide. A survey that assessed the prevalence, molecular characteristics, and zoonotic potential of these pathogens was conducted on a variety of dogs in Guangzhou, southern China. A total of 651 canine stool samples from household (*n* = 199), shelter (*n* = 149), breeding (*n* = 237), and pet market dogs (*n* = 66) were collected from eight districts in Guangzhou. *Cryptosporidium* spp.*, Giardia duodenalis*, and *Blastocystis* sp. were detected by PCR amplification of the *SSU* rRNA gene. *Giardia duodenalis-*positive specimens were further assigned into assemblages using the glutamate dehydrogenase gene*. Cryptosporidium* spp.,* G. duodenalis*, and *Blastocystis* sp. were found in 21 (3.2%), 20 (3.1%), and 35 (5.4%) samples, respectively. The overall prevalence of shelter dogs (40.28%, 60/149) was significantly higher than that of household (3.0%, 6/199), breeding (2.1%, 5/237), and pet market dogs (7.5%, 5/66) (χ^2^ = 154.72, df = 3, *P* < 0.001). Deworming in the past 12 months had a strong protective effect on the risk of contracting parasite infections (*P* < 0.001). No significant differences were detected between age or sex groups (*P* > 0.05). Dog-specific *C. canis* (*n* = 19) and zoonotic *C. parvum* (*n* = 2) were the only two *Cryptosporidium* species. Sequence analysis revealed the presence of three *G. duodenalis* assemblages: dog-specific assemblages D (*n* = 14) and C (*n* = 5), and cat-specific F (*n* = 1). Zoonotic *Blastocystis* ST3 (*n* = 28) was the dominant subtype, followed by ST1 (*n* = 6) and ST10 (*n* = 1). To our knowledge, this is the first large-scale investigation on the occurrence and molecular characteristics of *Blastocystis* sp. in dogs in China. Our results indicated that the dogs seemed to play a negligible role as reservoirs for *Cryptosporidium* spp. and *G. duodenalis* transmission to humans, but they are potential novel suitable hosts of *Blastocystis* sp. A strict sentinel surveillance system of dogs should be established to minimise the zoonotic risk of spreading blastocystosis among humans and dogs.

## Introduction

*Cryptosporidium* spp., *Giardia duodenalis*, and *Blastocystis* sp. are cosmopolitan enteric protists with a wide range of hosts, including humans, non-human primates, companion animals, ruminants, birds, and wild mammals^1–3^. Although the pathogenicity of *Blastocystis* sp. is under strong debate, it may also be associated with gastrointestinal disease-causing agents like *Cryptosporidium* spp*.* and *G. duodenalis*, which cause issues such as self-limiting diarrhoea, abdominal pain, irritable bowel syndrome, and flatulence^1,3–5^. In particular, people with compromised immune systems (e.g. AIDS patients and organ transplant recipients) are susceptible to these infections^1,6,7^. Infections occur mainly by faecal–oral transmission after ingestion of infective forms (oocysts or cysts), usually via water, food, or direct contact^1,3,8^. *Cryptosporidium parvum, C. hominis*, *C. meleagridis*, *C. canis*, and *C. muris* are the five most common human pathogenic species of *Cryptosporidium*, of which *C. canis* is the most prevalent species in dogs^3,9–11^. *G. duodenalis* consists of eight distinct assemblages or genotypes (A–H). Assemblages A and B have a wide host range and are responsible for the majority of known human disease cases, whereas assemblages C–H seem to be host-specific for non-human species^1^. Dogs are predominantly infected by assemblages C and D^1,12–14^. Additionally, at least 17 distinct *Blastocystis* subtypes were identified. ST1–9 and ST12 are the common zoonotic subtypes, and ST10–11 and ST13–17 only infect non-human species^15^.

Recently, a few molecular epidemiological surveys of *Cryptosporidium* spp*.*, *G. duodenalis*, and *Blastocystis* sp. in dogs have been conducted worldwide, and numerous species/assemblages/subtypes have been detected, such as *C. canis*, *C. parvum*, *C. muris*, *C. meleagridis*, *C. hominis*, *G. duodenalis* assemblages A–F, and *Blastocystis* ST1–ST6 and ST10^10,14^ (Tables 1, 2, 3). However, little information on *Blastocystis* sp. infection and subtype distribution in dogs in China is available^16^. Additionally, only one pathogen was involved in most of these studies.

**Table 1:** Summary of data from studies of *Cryptosporidium* spp. in dogs worldwide, 2010–2020.

Location	Period	Methods	No. examined	No. positive (%)	Populations	Species/assemblages/subtypes (*n*)	References
China	2015	PCR	484	24 (4.9)	pet	*C. canis* (20)	^14^
China	2011–2014	PCR	485	39 (8.0)	household, pet	*C. canis* (39)	^11^
Nigeria	2017	PCR	203	5 (2.5)	free-ranging	*C. parvum* (3), *C. muris* (2)	^17^
China	2013–2014	PCR	267	6 (2.2)	pet, stray	*C. canis* (5), *C. ubiquitum* (1)	^18^
China	2017–2018	PCR	641	44 (6.9)	pet	*C. canis* (42), *C. muris* (1), *C.* rat genotype IV (1)	^19^
France	2012–2013	PCR	116	3 (2.6)	household	*C. canis* (3)	^20^
Japan	2011–2012	PCR	1096	111 (10.1)	household, pet	*C. canis* (111)	^21^
Japan	2014–2017	PCR	314	66 (21.0)	breeding kennel	*C. canis* (66)	^22^
Spain	2013–2016	DFM, PCR	194	8 (4.1)	sheltered	*C. canis* (5), *C. hominis* (1)	^23^

*DFM* direct fluorescence microscopy.

**Table 2:** Summary of data from studies of *G. duodenalis* in dogs worldwide, 2010–2020.

Location	Period	Methods	No. examined	No. positive (%)	Populations	Species/assemblages/subtypes (*n*)	References
China	2015	PCR	484	62 (12.8)	Pet	D (15), C (5), F (1)	^14^
China	2010–2011	PCR	209	23 (11)	Pet	D (18), A (5)	^12^
China	2016–2017	PCR	527	57 (10.8)	Stray	A (26), C (18), D (13)	^13^
China	2011–2014	PCR	485	127 (26.2)	Household, pet	A (23), B (1), C (26), D (58)	^11^
Spain	2014–2016	DFM, qPCR	348	127 (36.5)	Sheltered, breeding, hunting, shepherd, pet	A (5), B (8), C (2), D (13)	^24^
China	2013–2014	PCR	267	12 (4.5)	Pet and stray	C (7), E (5)	^18^
China	2011	PCR	205	27 (13.2)	Police and farm	A (25), C ( 2)	^25^
China	2017–2018	PCR	641	60 (9.4)	Pet	C (27), D (26)	^19^
Korea	2017–2018	PCR	640	99 (15.5)	Sheltered, companion, special purpose	C (16), D (24)	^26^

*DFM* direct fluorescence microscopy.

**Table 3:** Summary of data from studies of *Blastocystis* sp*.* in dogs worldwide, 2010–2020.

Location	Period	Methods	No. examined	No. positive (%)	Populations	Species/assemblages/subtypes (*n*)	References
China	2015–2017	PCR	136	4 (2.9)	Peand farm	ST1 (3), ST4 (1)	^16^
USA	2012	PCR	103	10 (9.7)	Sheltered, client-owned	ST1 (2), ST10 (2)	^27^
Philippines	2011–2012	PCR	145	23 (15.8)	Pet	ST1 (1), ST2 (2), ST3 (4), ST4 (3), ST5 (3), unkown (10)	^28^
India	2010–2011	PCR	80	19 (24)	Stray	ST1 (9), ST4 (2), ST5 (1), ST6 (7)	^29^
Australia	2010–2011	PCR	80	2 (2.5)	Pet and pound	ST1 (2)	^29^
Cambodia	2010–2011	PCR	80	1 (1.3)	Semi-domestic	ST2 (1)	^29^
Brazil	2011–2013	PCR	49	0 (0)	Pet	–	^30^
Brazil	2018	PCR	20	0 (0)	Pet	–	^31^
Brazil	2013–2014	CM	78	2 (2.6)	Domestic	–	^32^
Colombia	2013–2014	PCR	40	15 (37.5)		ST2 (15)	^33^
Spain	2014	PCR	55	0 (0)		–	^34^
Turkey	2010	PCR	–	–	–	ST1 (1), ST2 (3)	^35^
France	2012–2013	PCR	116	4 (3.4)	Household	ST2 (2), ST10 (2)	^20^

*CM* conventional microscopy.

As intimate companions, dogs have close contact with humans. However, dogs often harbour intestinal protozoa, which can cause mild to severe disease in dogs and lead to zoonotic infections in humans. Among these protozoa, *G. duodenalis*, *Cryptosporidium* spp*.*, and *Blastocystis* sp. are common causes of diarrhoea in dogs worldwide. Guangzhou, southern China, is the third most economically developed city in China and boasts a large population of human residents (i.e. 14.90 million in 2017) and companion animals (i.e. > 10.62 million pets in the city in 2015) (Guangzhou Statistics Bureau; https://tjj.gz.gov.cn/). To date, one study of *Cryptosporidium* spp*.* and three molecular epidemiological studies of *G. duodenalis* have been published on dogs in the region^12,13,19,36^; however, nothing is known about the occurrence and molecular characterisation of *Blastocystis* sp*..* The purpose of our study was to estimate the overall occurrence of *Cryptosporidium* spp*., G. duodenalis*, and *Blastocystis* sp*.* in dogs living in areas of Guangzhou and assess the zoonotic potential between humans and dogs.

## Methods

### Study design

**Figure 1:**
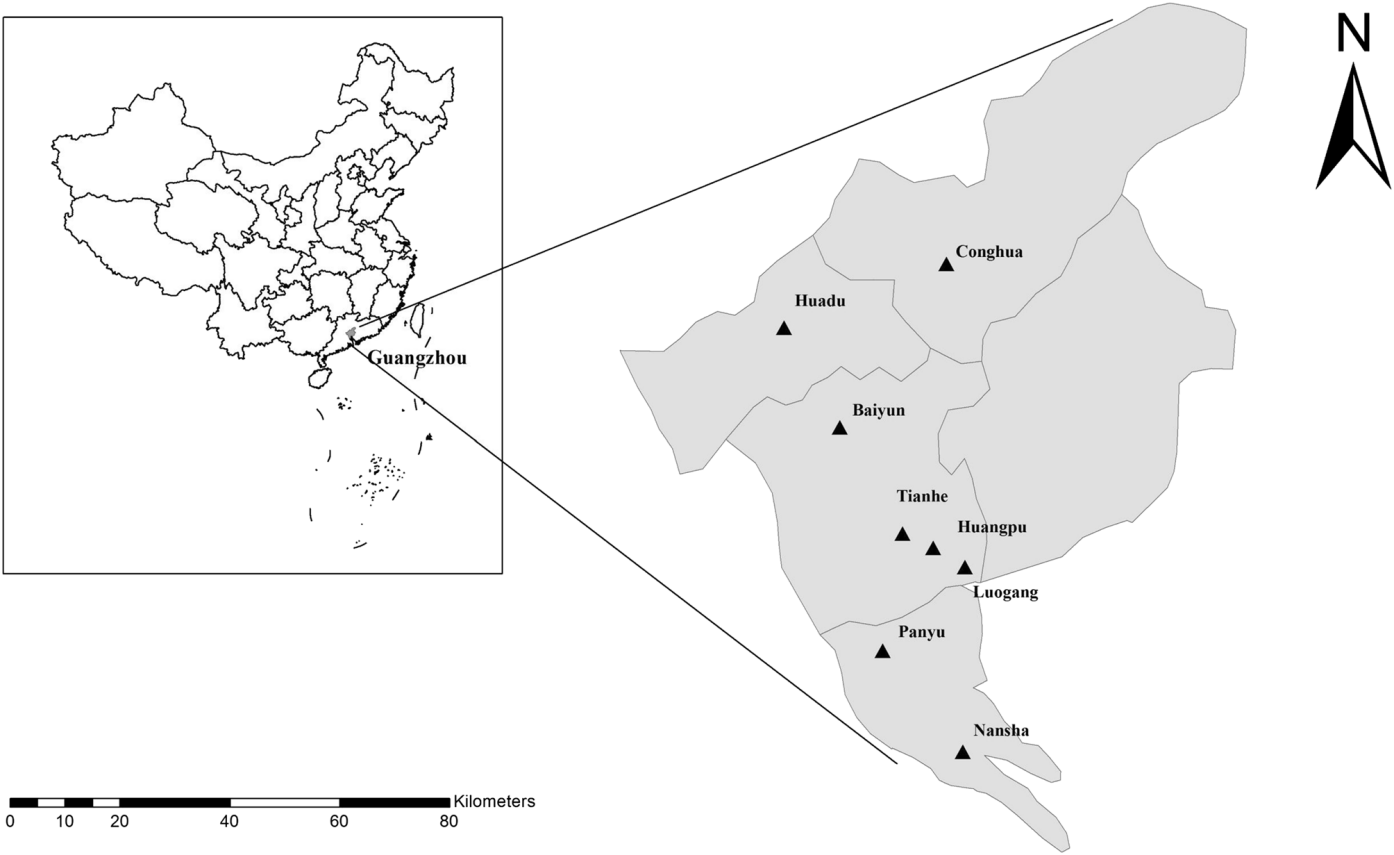
Geographic map of the sampling locations in this study. The figure was originally designed by the authors under the software ArcGIS 10.2. The original vector diagram imported in ArcGIS was adapted from Natural Earth (https://www.naturalearthdata.com).

The study was conducted in Guangzhou. Guangzhou is one of the largest metropolitan cities in southern China (coordinates 3° 28′–25° 31′ N and 108° 13′–119° 59′ E); it covers an area of 7434 m^2^ and has a population of about 140 million. The annual average temperature is 20–22 °C and the average relative humidity is 77% (Guangzhou Statistics Bureau; https://tjj.gz.gov.cn/). A total of 651 fresh faecal samples were randomly collected from 199 household dogs (from four pet hospitals located in four different districts in urban Guangzhou: Tianhe, Baiyun, Huadu, and Panyu Districts), 149 shelter dogs (from two shelters located in suburban Luogang and Huangpu Districts), 237 breeding dogs (from two breeding centres located in suburban Conghua and Nansha Districts), and 66 pet market dogs (from one pet market in urban Tianhe District), with or without a history of illness, on a single occasion between January and December 2018 (Fig. 1). Faecal samples from shelters, breeding centres, and pet markets were collected as soon as practicably possible after defaecation by our research staff, either directly from the floor of the cage or per rectum. Care was taken to avoid sampling faecal material that had contacted the ground at the time of sampling. All samples from household dogs were collected immediately after natural defecation and donated by the dog owners, who provided consent for the use of samples from their animals in the survey. All collected fresh samples had no apparent diarrhoeal symptoms at the time of sampling. The samples were placed into clean plastic bags marked with ID numbers corresponding to the date, origin, age, sex, and whether the dog was dewormed in the past 12 months. The plastic bags were sealed and immediately placed onto ice packs in an insulated container. Samples were transported to the laboratory, stored at 4 °C, and processed no later than 24 h after collection.

### DNA extraction

A 10-g aliquot of each sample was individually mixed with 30 ml of distilled water and passed through a ~ 250-μm-wide wire mesh sieve. Suspensions were centrifuged at 3000 × *g* for 5 min and precipitates were used for DNA extraction. Genomic DNA was extracted from 200 mg of each precipitate using an E.Z.N.A. Stool DNA Kit (Omega Bio-Tek Inc., Norcross, GA, USA) according to the manufacturer’s instructions. To improve the quality of recovered DNA, the mixtures of stool samples and SLX-Mlus buffers were vibrated at maximum speed for 15 min until the stool samples were thoroughly homogenised. Extracted DNA was stored at − 20 °C.

### PCR detection

*Cryptosporidium* spp*.* was detected by nested PCR amplification of an approximately 830-bp fragment of the *18S* rRNA gene as previously described^37^. *G. duodenalis* was detected by nested PCR amplifications of a 290-bp product of the *18S* rRNA gene and a 520-bp polymorphic fragment of the glutamate dehydrogenase (*GDH*) gene as described^38,39^. To detect *Blastocystis* sp*.*, an approximately 600-bp fragment of the *18S* rRNA gene was amplified by single primer PCR as previously described (see Supplementary Table S1 online)^40^. Each 25 μl of PCR mixture contained 0.4 μM of each primer, 2.5 μl 10 × *Taq* Buffer (Mg^2+^ free; GC Buffer for the *G. duodenalis 18S* rRNA gene), 2 mM MgCl_2_, 0.2 mM dNTP mixture, 0.625 U of TaKaRa *Taq* (TaKaRa Shuzo Co., Ltd., Otsu, Japan), and 1 μl of genomic DNA. Each specimen was analysed in duplicate using positive (cattle-derived DNA) and negative (sterile water) controls.

### Sequence and phylogenetic analysis

The positive secondary PCR products were directly sequenced by GENEWIZ (Suzhou, China). Sequence accuracy was confirmed with two-directional sequencing. All raw sequencing data were viewed and aligned by eye in Chromas Pro 1.33 (Technelysium Pty. Ltd., Helensvale, Queensland, Australia). The identity of species/assemblages/subtypes was established by comparing the obtained sequences with reference sequences from the National Center for Biotechnology Information (https://www.ncbi.nlm.nih.gov/) database using Clustal X 2.1 (https://www.clustal.org). Phylogenetic analysis was performed by a neighbour-joining (NJ) analysis in MEGA 7.01 (https://www.megasoftware.net/) based on the Kimura 2-parameter model using 1000 bootstrap replicates. The *p*-distance model was selected as the most suitable model.

### Statistical analysis

Differences between prevalence and the dog’s origin (households, pet market, breeding centres, and shelters), age (≤ 6 months vs. > 6 months), sex, and deworming conditions (dewormed vs. non-dewormed in the past 12 months) were compared using a χ^2^ test in SPSS 22.0 for Windows (SPSS Inc., Chicago, IL, USA) with 95% confidence intervals. Differences at *P* < 0.01 were considered significant.

### Statement of informed consent, ethics approval and guidelines

Prior to fecal specimen collection, we showed an informed consent to the dogs owner. This informed consent provides some information, including the purpose of the study, research approval number, the benefits and risks that may bring to their animals participating in the study. The informed consent was obtained from the dog owners. Appropriate permission in written form was obtained from the animal owners. During specimen collection, all animal work strictly followed the guidelines relating to the recommendations from the Guide for the Care and Use of LaboratoryAnimals of the Ministry of Health, China. Our protocol in written form was authorized by the Animal Ethics Procedures and Guidelines of the People's Republic of China and the approval of China Guangdong Province Science and Technology Department (Permit Number: SYXK (Yue) 2011–2018).

## Results

### Overall prevalence of *Cryptosporidium* spp*.*, *G. duodenalis*, and *Blastocystis* sp.

Faecal samples from 651 dogs were tested by PCR for the presence of *Cryptosporidium* spp*.*, *G. duodenalis*, and *Blastocystis* sp. The prevalence and 95% confidence intervals are summarised in Table 4. *Cryptosporidium* spp*.*, *G. duodenalis*, and *Blastocystis* sp. were detected in 21 (3.2%), 20 (3.1%), and 35 (5.4%) of the examined samples, respectively. Co-infection rates significantly differed based on the dogs’ origins. The overall prevalence of shelter dogs (40.28%, 60/149) was significantly higher than that of household (3.0%, 6/199), breeding (2.1%, 5/237), and pet market dogs (7.5%, 5/66) (χ^2^ = 154.72, *df* = 3, *P* < 0.001). Moreover, deworming in the past 12 months had a strong protective effect on the risk of contracting pathogens, with non-dewormed dogs having 15-times higher risk of overall positive infection rates than dewormed animals (χ^2^ = 101.92, *df* = 1, *P* < 0.001). No significant difference was observed between age categories (≤ 6-month-old puppies and > 6-month-old young animals) in the prevalence of *Cryptosporidium* spp. (3.8% in puppies vs. 2.6% in young animals; χ^2^ = 0.69, *P* = 0.406), *G. duodenalis* (4.1% in puppies vs. 2.0% in young animals; χ^2^ = 2.40, *P* = 0.122), or *Blastocystis* sp. (3.8% in puppies vs. 7.2% in young animals; χ^2^ = 3.73, *P* = 0.053). There were also no significant differences in overall (9.1% in male dogs vs. 14.8% in female dogs; χ^2^ = 5.04, *P* = 0.025) and pathogen-specific prevalence rates between the two sex groups of dogs (χ^2^ = 3.49 for *G. duodenalis*, χ^2^ = 0.03 for *Cryptosporidium* spp., and χ^2^ = 3.58 for *Blastocystis* sp.; *df* = 1, *P* > 0.05).

**Table 4:** Risk factors analysis on the prevalence of *Cryptosporidium, G. duodenalis* and *Blastocystis* in dogs.

Factor	Category	No. tested	No. of positive (positive rate %; CI95)			Co-infection	*p*
			*G. duodenalis*	*Cryptosporidium*	*Blastocystis*		
Origin	Shelters	149	13 (8.7; 7.0–10.4)	12 (8.1; 6.4–9.8)	35 (23.5; 21.4–25.6)	60 (40.2; 3.9–42.5)	< 0.001
	Households	199	2 (1.0; 0.2–1.8)	4 (2.0; 1.0–3.0)	0	6 (3.0; 1.9–4.1)	
	Pet market	66	5 (7.5; 2.2–7.8)	0	0	5 (7.5; 2.2–7.8)	
	Breeding centers	237	0	5 (2.1; 1.2–3.0)	0	5 (2.1; 1.2–3.0)	
Age	≤ 6 month	345	14 (4.1; 3.3–4.9)	13 (3.8; 3.0–4.6)	13 (3.8; 3.0–4.6)	40 (11.6; 10.6–12.6)	0.946
	> 6 month	306	6 (2.0; 1.3–2.7)	8 (2.6; 1.8–3.4)	22 (7.2; 6.2–8.2)	36 (11.8; 10.7–12.9)	
Sex	Male	361	7 (1.9; 1.3–2.5)	12 (3.3; 2.6–4.0)	14 (3.9; 3.2–4.6)	33 (9.1; 8.2–10.0)	0.025
	Female	290	13 (4.5; 3.6–5.4)	9 (3.1; 2.3–3.9)	21 (7.2; 6.2–8.2)	43 (14.8; 13.6–16.0)	
Deworming	Yes	436	5 (1.1; 0.6–1.6)	7 (1.6, 1.1–2.1)	0	12 (2.8; 12.0–13.6)	< 0.001
	No	215	15 (7.0, 5.7–8.3 )	14 (6.5%; 5.3–7.7)	35 (16.3; 14.8–17.8)	64 (30.0; 28.3–31.7)	
Total		651	20 (3.1; 2.6–3.6)	21 (3.2; 2.7–3.7)	35 (5.4; 4.9–5.9)	76 (11.7; 11.1–12.3)	

### *Cryptosporidium* species

Sequence analysis of the 21 *SSU* rRNA *Cryptosporidium*-positive canine samples revealed the presence of *C. canis* (*n* = 19) and *C. parvum* (*n* = 2) (Table 5). Nucleotide sequences of *C. canis* and *C. parvum* detected in this study had 100% similarity to those deposited sequences in GenBank under accession numbers EU754826 and MF074695, respectively.

**Table 5:** Species/assemblages/subtypes of *Cryptosporidium*, *G. duodenalis* and *Blastocystis* in dogs.

Factor	Category	No. tested	Assemblages/species/subtypes/(no. of specimens)		
			*G. duodenalis*	*Cryptosporidium*	*Blastocystis*
Site	Shelters	149	D (11), C (2)	*C. canis* (11), *C. parvum* (1)	ST3 (28), ST1 (6), ST10 (1)
	Households	199	D (1), F (1)	*C. canis* (4)	–
	Pet market	66	D (2), C (3)	–	–
	Breeding centers	237	0	*C. canis* (4), *C. parvum* (1)	–
Age	≤ 6 months	345	D (8), C (5), F (1)	*C. canis* (12), *C. parvum* (1)	ST3 (11), ST1 (2)
	> 6 months	306	D (6)	*C. canis* (7), *C. parvum* (1)	ST3 (17), ST1 (4), ST10 (1)
Sex	Male	361	D (5), C (1), F (1)	*C. canis* (12)	ST3 (10), ST1 (3), ST10 (1)
	Female	290	D (9), C (4)	C. *canis* (7), *C. parvum* (2)	ST3 (18), ST1 (3)
Deworming	Yes	436	D (4), C (1),	*C. canis* (7)	–
	No	215	D (10), C (4), F (1)	*C. canis* (12),*C. parvum* (2)	ST3 (28), ST1 (6), ST10 (1)
Total		651	D (14), C (5), F (1)	*C. canis* (19),*C. parvum* (2)	ST3 (28), ST1 (6), ST10 (1)

### *G. duodenalis* assemblages

**Figure 2:**
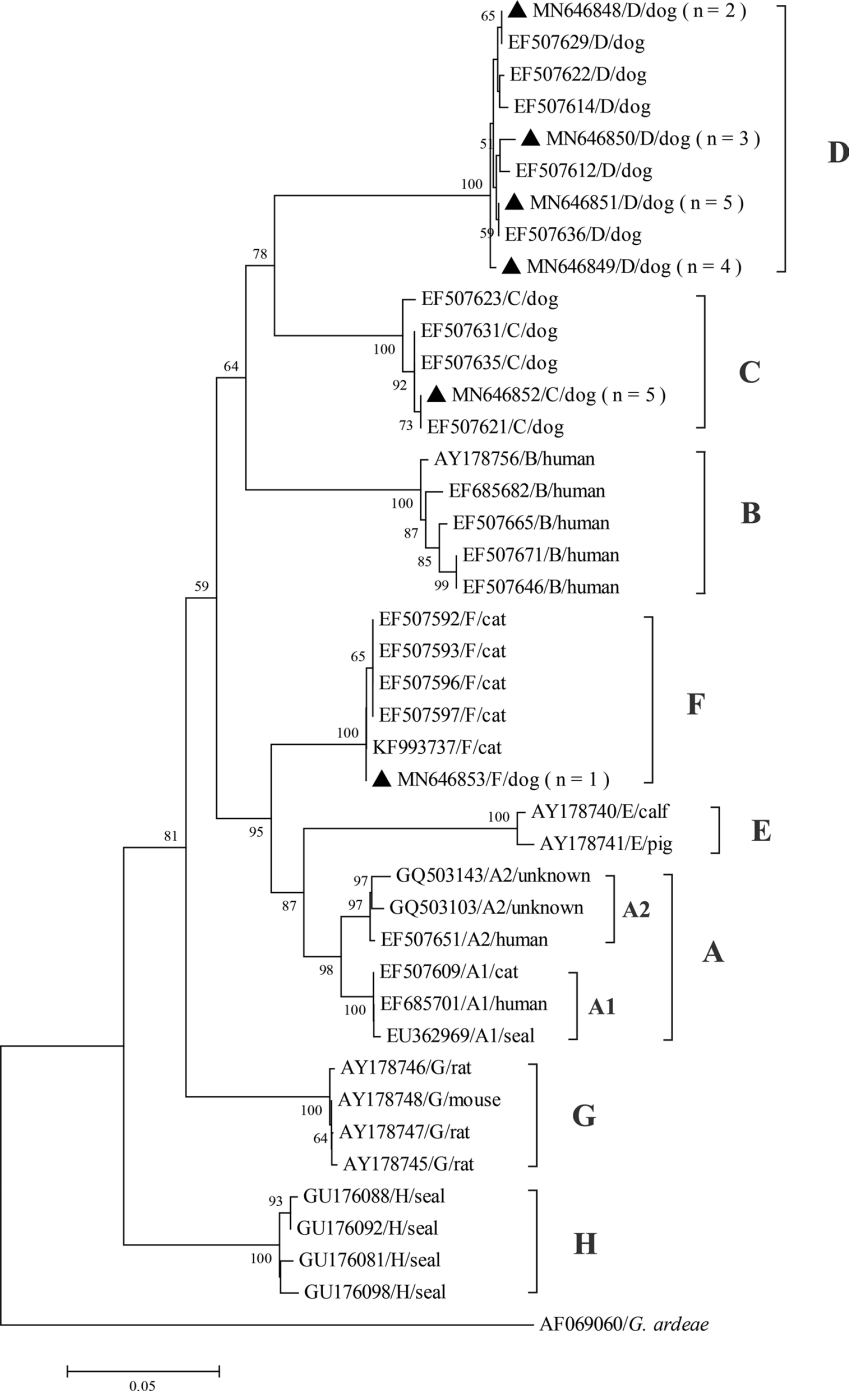
Phylogenetic tree depicting evolutionary relationships among assemblages of *G. duodenalis* at the *gdh* locus using the Neighbor-Joining analysis. Bootstrap values lower than 50% were not displayed. Filled circles represent canine sequences from this study. Giardia ardeae was used as outgroup taxa.

Sequence analysis revealed the presence of three different *G. duodenalis* assemblages: D (*n* = 14), C (*n* = 5), and F (*n* = 1). Three SSU rRNA nucleotide sequences of *G. duodenalis* assemblages D, C, and F were 100% identical to the GenBank reference sequences DQ385549, DQ385548, and JX275387, respectively. The genotypes identified by the *GDH* gene were fully consistent with those identified by the *SSU* rRNA gene and there were no overlapping nucleotides at any position, as shown by the chromatograms. No mixed assemblage infections were identified (see Supplementary Table S2 online). Moreover, four *GDH* sub-assemblage D nucleotide sequences were identified in this study. One sub-assemblage D sequence was identical to GenBank EF507636 reference sequences (*n* = 5), and the remaining three sequences had minor differences from EF507636, including two single nucleotide polymorphism (SNPs) in four specimens (C to T substitution at position 162 and T to G substitution at position 324), three SNPs in two specimens (C to T substitution at position 162, A to G substitution at position 174, and A to T substitution at position 311), and three SNPs in three specimens (T to C substitution at position 109, A to G substitution at position 183, and A to T substitution at position 312). All GDH sub-assemblage C sequences were identical to each other and showed 99% sequence similarity to the EF507621 reference sequence, with one SNP difference at position 12 (T → C). The assemblage F nucleotide sequence was identical to the corresponding KF993737 sequence of from a cat in China (Fig. 2).

### *Blastocystis* subtypes

DNA sequencing of the *SSU* rRNA PCR products from the 35 *Blastocystis*-positive samples and sequence analysis indicated the existence of subtypes ST3 (*n* = 27), ST1 (*n* = 6), ST10 (*n* = 1), and unknown ST (*n* = 1). Twenty-seven nucleotide sequences identified as ST3 were identical to each other and had 100% similarity to the MK782518 reference sequence from urticaria patients in Brazil. Six ST1 nucleotide sequences included two different nucleotide sequences with 100% similarity to the GenBank reference sequences MK782501 in four specimens and MK782521 in two specimens. The ST10 nucleotide sequence had 100% similarity to a cattle-derived sequence from Malaysia in GenBank (MK240480). The remaining nucleotide sequence was not assigned a subtype by the sequence typing database, and was 100% homologous with the published sequence MK511788 from Malaysia.

**Figure 3:**
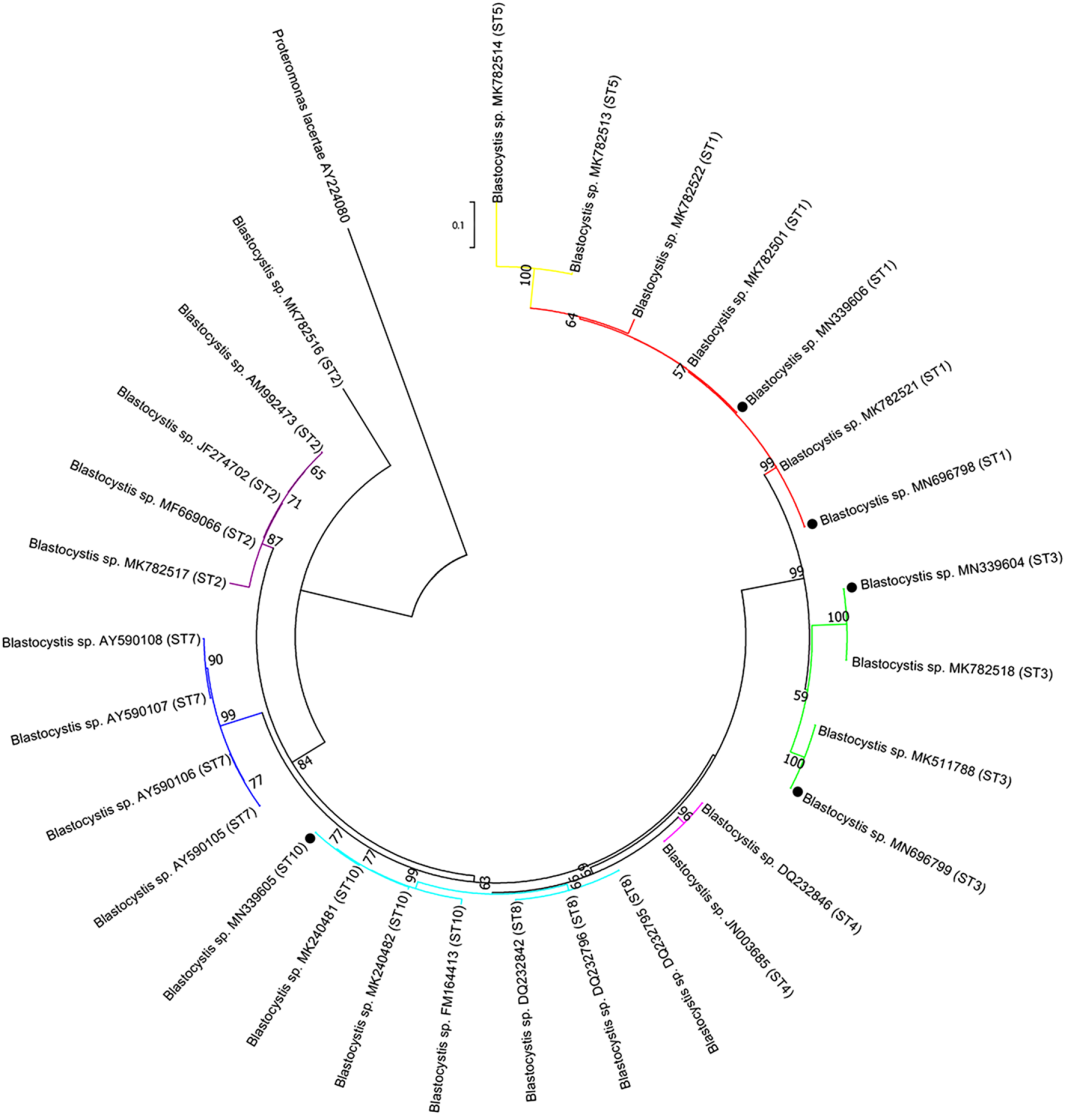
Bootstrap consensus phylogenetic tree for aligned small subunit rDNA sequences from *Blastocystis* spp. isolated from canines and previously published representative sequences, using *Proteromonas lacerate* as an outgroup. The tree was derived using the neighbor-joining method based on the Kimura 2-parameter model with 1000 bootstrap replicates. Taxa isolated from canine samples are shown by filled circles.

Phylogenetic analysis using NJ analyses clustered *Blastocystis* subtypes obtained in the present study into three subtypes (ST1, ST3, and ST10), and the unknown subtype was grouped into subtype ST1 (Fig. 3).

## Discussion

In this study, *Cryptosporidium* spp., *G. duodenalis*, and *Blastocystis* sp. were found at considerably low prevalences (3.2%, 3.1%, and 5.4%, respectively) in the surveyed canine populations. This finding is comparable to the prevalences in Poland (2.0% for *G. duodenalis*)^41^, India (3.0% for *G. duodenalis*)^42^, the United States (2.0% and 3.8% for *Cryptosporidium*)^43,44^, China (3.8% and 4.9% for *Cryptosporidium*)^10,14^, Italy (3.3% for *Cryptosporidium*)^9^, Australia (2.5% for *Blastocystis*)^29^, Brazil (2.6% for *Blastocystis*)^31^, and France (3.4% for *Blastocystis*)^20^. However, higher prevalences of 21% of *Cryptosporidium* from Japan, 36.5% of *G. duodenalis* from Spain, and 37.5% of *Blastocystis* from Colombia were also previously found in dogs^22,24,33^. Aside from geographical considerations, many factors can contribute to this difference in the prevalence, including a dog’s age, origin, health status, and examination methods used. Importantly, the true prevalences may be underestimated because of the intermittent shedding of oocysts/cysts, low parasitic burdens, and invalid amplification^23^. Therefore, it is important to perform a well-designed longitudinal study that includes appropriate sampling methods and a combination of diagnostic tests (e.g. microscopic examination, antigen assay, and PCR assay) to estimate the real prevalence.

In risk factor analysis, the overall and pathogen-specific prevalences of shelter dogs were significantly higher than those of household, breeding, and pet market dogs. Poor care conditions may be a significant factor that contributed to the high prevalence in shelter dogs^23^. Additionally, these shelter dogs originally roamed free in the nearby streets before they were found by local citizens and sent to the shelters, which increased their exposure to a variety of pathogens. No significant age- and sex-associated differences were detected in the prevalence of these three pathogens, which is consistent with observations of previous studies in China, Japan, Colorado, France^11,20,21,44^ , and Australia^10,12,45^. As expected, deworming had a significant negative effect on the risk of overall and single pathogen infections. This could be related to the fact that the anthelminthic ingredients used for dogs in China mainly include ivermectin, pyrantel, praziquantel, pyrantel pamoate, febantel, nitazoxanide, metronidazole, and fenbendazole, most of which are also effective against protozoan infections, such as *Giardia* spp., *Cryptosporidium* spp., and *Blastocystis* sp.^46–48^. Thus, pet hygiene management is suggested to be a major risk factor for contracting these pathogens in dogs.

The sequencing data revealed that dogs were predominantly infected by the expected host-specific species *C. canis*. Another interesting outcome was the identification of *C. parvum* in two of the canine isolates genotyped. *Cryptosporidium parvum* is the most frequent species known in cryptosporidium infections of humans and has resulted in several zoonotic outbreaks^49^. However, because of the omnivorous nature of dogs, *C. parvum* detected from dogs in this study may have been present because of accidental acquisition or mechanical carriage of *C. parvum* oocysts of anthroponotic origin via environmental contamination. Although the host-specific species *C. canis* also colonised individuals in hospitals, including children, HIV patients, and even immunocompetent individuals^50–52^, the infections in humans were likely transient^53^. Based on our data and that of other studies, we conclude that dogs do not seem to be suitable reservoirs for *Cryptosporidium* spp. transmission to humans, therefore, posed a limited risk to humans. This is consistent with the findings reported in dog populations in eastern Spain, where most of the genotypes identified seemed to be primarily transmitted within canine cycles^24^.

Regarding *G. duodenalis*, dogs were infected by assemblages D and C. Surprisingly, the supposedly cat-specific assemblage F was also found in one household dog, which was consistent with a previous report in Beijing, China^14^. Considering there was little possibility of the specimen being contaminated with cat faeces, as individual faecal samples were freshly collected by pet dog owners who kept only a single companion animal, it is more likely that the dog was transiently infected by ingesting parasite cysts of cat origin. Assemblages D and C have strong host specificities and have been mainly detected in canines. Both are considered of limited zoonotic relevance, although sporadic cases of human infections have been frequently detected in travellers, children, and diarrhoeal outpatients^53–55^. The dominant appearances of *G. duodenalis* assemblages D and C in dogs in our study suggested that zoonotic transmission of giardiasis rarely occurs between humans and dogs.

A high diversity of *Blastocystis* subtypes has been identified in dogs worldwide and the subtype constitution was observed to differ among geographical regions, such as ST1 and ST4 in China; ST1, ST4, ST5, and ST6 in India; ST1 and ST10 in the USA; ST1, ST3, and ST4 in Australia; and ST1 and ST2 in Thailand^27,29,35,56,57^. This is the first large-scale survey on the prevalence and genetic characteristics of *Blastocystis* sp. in dogs of various origins in China, and we detected the presence of subtypes ST3, ST1, and ST10. Among the subtypes in this study, ST3 and ST1 are the two predominant subtypes. The subtype distribution of *Blastocystis* in dogs in our study consistent with most populations in humans around the world^15,57,58^. In China, in a cross-sectional survey in humans from four epidemiological settings, ST3 was the predominant type, accounting for 60.4% (*n* = 116) of 192 positive specimens, followed by subtype ST1, accounting for 24.5% (*n* = 47)^58^. A report from Argentina also showed that *Blastocystis* ST3 was the most prevalent subtype (48 cases) among 76 patients infected with *Blastocystis*, and other subtypes identified were ST1 (14.9%), ST6 (7.5%), and ST2 (5.9%)^57^. Moreover, ST3 and ST1 were found in dogs and their owners in Australia, the Philippines, and Turkey^15,35,59^. All of these studies suggested that ST3 and ST1 might be the important sources of human-to-human or animal-to-human transmission of *Blastocystis*, although more environmental factors and or other animal sources should be included. *Blastocystis* ST10 was found in one dog sample; this subtype is frequently identified in common livestock, including cattle, sheep, goats, and deer^3,16,60^, and occasionally found in wild animals, pigs, dogs, and cats^20,27^. Taken together, our results revealed that canines are a novel reservoir for *Blastocystis.*

We conducted partial assessment of the zoonotic potential of our canine isolates using only molecular epidemiological data, because assessing the risk for zoonotic transmission of these pathogens from dogs to/from humans is difficult. The only way to properly determine zoonotic transmission is by conducting case–control studies that assess the genotypes/subtypes of these pathogens by using appropriate molecular typing tools in human and canine populations that maintain permanent close contact in the same spatial and temporal setting^23,34,61,62^. However, our epidemiological study still generated baseline information and determined the genetic diversity of these pathogens in the investigated region.

## Conclusions

The prevalence and molecular characteristics of *Cryptosporidium* spp*.*, *G. duodenalis*, and *Blastocystis* sp. were determined in dogs in Guangzhou. Our risk factor analysis showed that management of pet hygiene may be a major risk factor for contracting these pathogens in dogs. Our results suggest that dogs do not seem to be suitable reservoirs of human giardiasis or cryptosporidiosis in the investigated region, but may act as novel suitable hosts of human blastocystosis. Strict sentinel surveillance of dogs, especially stray dogs, should be established to minimise the risk of spreading blastocystosis among humans and dogs.

## Supplementary information

Supplementary Table S1.

Supplementary Table S2.

## Data Availability

The datasets analyzed during the current study are available in the NCBI GenBank repository (https://www.ncbi.nlm.nih.gov/genbank/) under accession numbers: MN646215–MN646217 and MN646848–MN646853 for *G. duodenalis*, MN696800–MN696801 for *Cryptosporidium*, and MN339604–MN339606 and MN696798–MN696799 for *Blastocystis*.
