# Supplementary material for: Occurrence and genotypes of Cryptosporidium spp., Giardia duodenalis, and Blastocystis sp. in household, shelter, breeding, and pet market dogs in Guangzhou, southern China
Source: Sci Rep. 2020 Oct 20;10:17736. doi: 10.1038/s41598-020-74299-z (PMC7576217; doi:10.1038/s41598-020-74299-z)
Supplement: Supplementary file 1 — Supplementary Table S1. [file 41598_2020_74299_MOESM1_ESM.docx]

**Occurrence and genotypes of *Cryptosporidium* spp., *Giardia duodenalis*, and *Blastocystis* sp. in household, shelter, breeding, and pet market dogs in Guangzhou, southern China**

Liao Shenquan^1#^, Lin Xuhui^1#^, Sun Yongxiang^1,2^, Qi Nanshan^1^, Lv Minna^1^, Wu Caiyan^1^, Li Juan^1^, Hu Junjing^1^, Yu Linzeng^1^, Cai Haiming^1^, Xiao Wenwan^1^, Sun Mingfei^1^*, Li Guoqing^2^*

**Table S1**. **Target, primers, amplicon size and annealing temperature of the loci used for *G. duodenalis*, *Cryptosporidium* and *Blastocystis*.**

|  | gene | Primer (sequence 5´–3´) | Fragment length (bp) | Annealing  temperature (℃) | References |
| --- | --- | --- | --- | --- | --- |
| *G. duodenalis* | *SSU* rRNA | Gia2029 (AAGTGTGGTGCAGACGGACTC) | 292 | 55 |  |
|  |  | Gia2150c  (CTGCTGCCGTCCTTGGATGT) |  |  |  |
|  |  | RH11  (CATCCGGTCGATCCTGCC) |  | 59 |  |
|  |  | RH4 (AGTCGAACCCTGATTCTCCGCCCAGG) |  |  |  |
|  | *gdh* | ghd1  (TTCCGTRTYCAGTACAACTC) | 520 | 50 |  |
|  |  | gdh2  (ACCTCGTTCTGRGTGGCGCA) |  |  |  |
|  |  | gdh3 (ATGACYGAGCTYCAGAGGCACGT) |  | 50 |  |
|  |  | gdh4  (GTGGCGCARGGCATGATGCA) |  |  |  |
| *Cryptosporidium* | *SSU* rRNA | F1  (TTCTAGAGCTAATACATGCG) | 830 | 55 |  |
|  |  | R1  (CCCATTTCCTTCGAAACAGGA) |  |  |  |
|  |  | F2 (GGAAGGGTTGTATTTATTAGATAAAG) |  |  |  |
|  |  | R2  (CTCATAAGG TGCTGAAGGAGTA) |  |  |  |
| *Blastocystis* | *SSU* rRNA | RD5  (ATCTGGTTGATCCTGCCAGT) | 600 |  |  |
|  |  | BhRDr (GAGCTTTTTAACTGCAACAACG) |  |  |  |
