# Supplementary material for: Occurrence and genotypes of Cryptosporidium spp., Giardia duodenalis, and Blastocystis sp. in household, shelter, breeding, and pet market dogs in Guangzhou, southern China
Source: Sci Rep. 2020 Oct 20;10:17736. doi: 10.1038/s41598-020-74299-z (PMC7576217; doi:10.1038/s41598-020-74299-z)
Supplement: Supplementary file 2 — Supplementary Table S2. [file 41598_2020_74299_MOESM2_ESM.docx]

**Occurrence and genotypes of *Cryptosporidium* spp., *Giardia duodenalis*, and *Blastocystis* sp. in household, shelter, breeding, and pet market dogs in Guangzhou, southern China**

Liao Shenquan^1#^, Lin Xuhui^1#^, Sun Yongxiang^1,2^, Qi Nanshan^1^, Lv Minna^1^, Wu Caiyan^1^, Li Juan^1^, Hu Junjing^1^, Yu Linzeng^1^, Cai Haiming^1^, Xiao Wenwan^1^, Sun Mingfei^1^*, Li Guoqing^2^*

**Table S2. Genotyping data of *G. duodenalis* isolates from pet dogs at the gdh and *SSU* rRNA genes.**

| Isolates | *gdh* | SSU rRNA |
| --- | --- | --- |
| SD63 | D4 | D |
| SD48 | D4 | D |
| SD8 | D2 | D |
| SD60 | D4 | D |
| SD27 | D4 | D |
| SD24 | D4 | D |
| SD38 | D3 | D |
| SD16 | D3 | D |
| SD6 | D3 | D |
| LZD3 | D2 | D |
| LZD17 | D2 | D |
| LZD38 | D2 | D |
| SD36 | D1 | D |
| SD18 | D1 | D |
| YD15 | C1 | C |
| LZD23 | C1 | C |
| LZD47 | C1 | C |
| LZD52 | C1 | C |
| LZD14 | C1 | C |
| YD91 | F1 | F |
